# Supplementary material for: Insulin and TOR signal in parallel through FOXO and S6K to promote epithelial wound healing
Source: Nat Commun. 2016 Oct 7;7:12972. doi: 10.1038/ncomms12972 (PMC5059774; doi:10.1038/ncomms12972)
Supplement: Supplementary Information — Supplementary Figures 1-14 and Supplementary Table 1 [file ncomms12972-s1.pdf]

SUPPLEMENTARY FIGURES

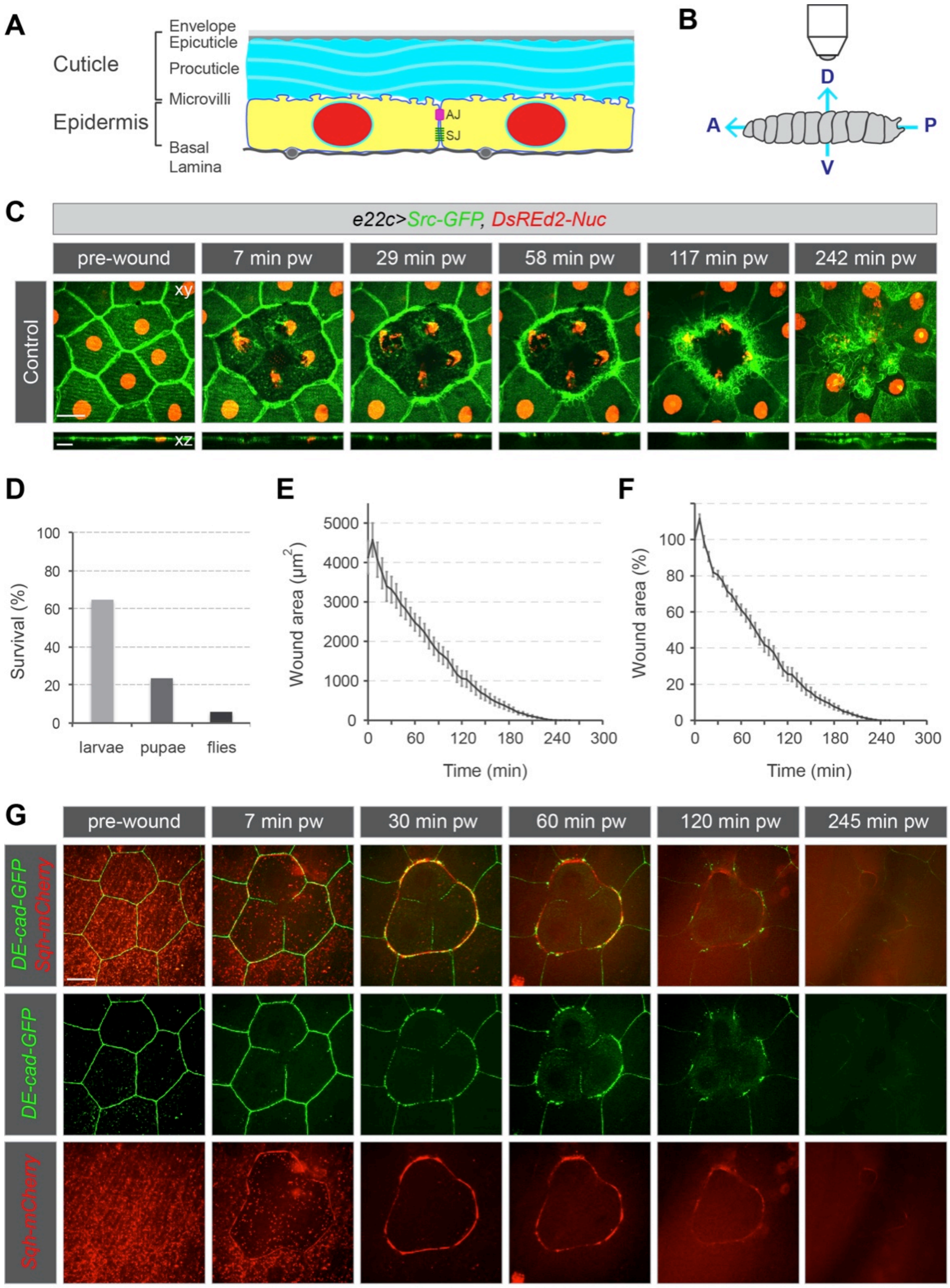

Supplementary Figure 1| Epidermal multi-cell wound healing. (**A**) Cartoon illustrating the structure of the larval epidermis. (**B**) Cartoon illustrating orientation of larvae for imaging. (**C-F**) Heterozygous *e22c>Src-GFP,DsRed2-Nuc (e22c-Gal4, UAS-Src-GFP, UAS-DsRed2-Nuc/+*) and (**G**) recombinant *DE-Cad-GFP, Sqh-mCherry* early third instar larvae were immobilised and wounded by laser. (**C-G**) Multi-cell wound healing (3-5 cells, wound area ~3,000-7,000 μm^2). (**C**) Projections (xy) and cross-sections (xz) of the wounded epidermis from a time-lapse series of a multi-cellular wound that was closed by 242 min. (**D**) Survival rates of larvae after wounding. After multi-cell wounding 65% of the larvae survived 48h, 24% pupated and 6% eclosed as adult flies. (**E-F**) Quantitative analysis of wound healing (n=9 larvae). Data given as means ± s.e.m.. (**E**) Changes in absolute wound area (μm^2) and (**F**) in relative wound area (%). After single-cell ablation, the wound area first expanded for 6±1 min. 50±4% of the wound area was covered within ~72 min and the remaining 50% closed within ~182 min. (**G**) Actomyosin dynamics of multi-cell wound healing of n=5 larvae. At 6±1 min an actomyosin cable started to form and was completed at 8-12 min and maintained throughout wound healing. AJ: adherens junction, SJ: septate junction, A: anterior, P: posterior, D: dorsal, V: ventral. Scale bars: (**C, G**) 20μm, (**C**) xz 10 μm. pw: post wounding, DE-Cad: Drosophila E-Cadherin, Sqh: spaghetti squash, the Drosophila non-muscle myosin II regulatory light chain.

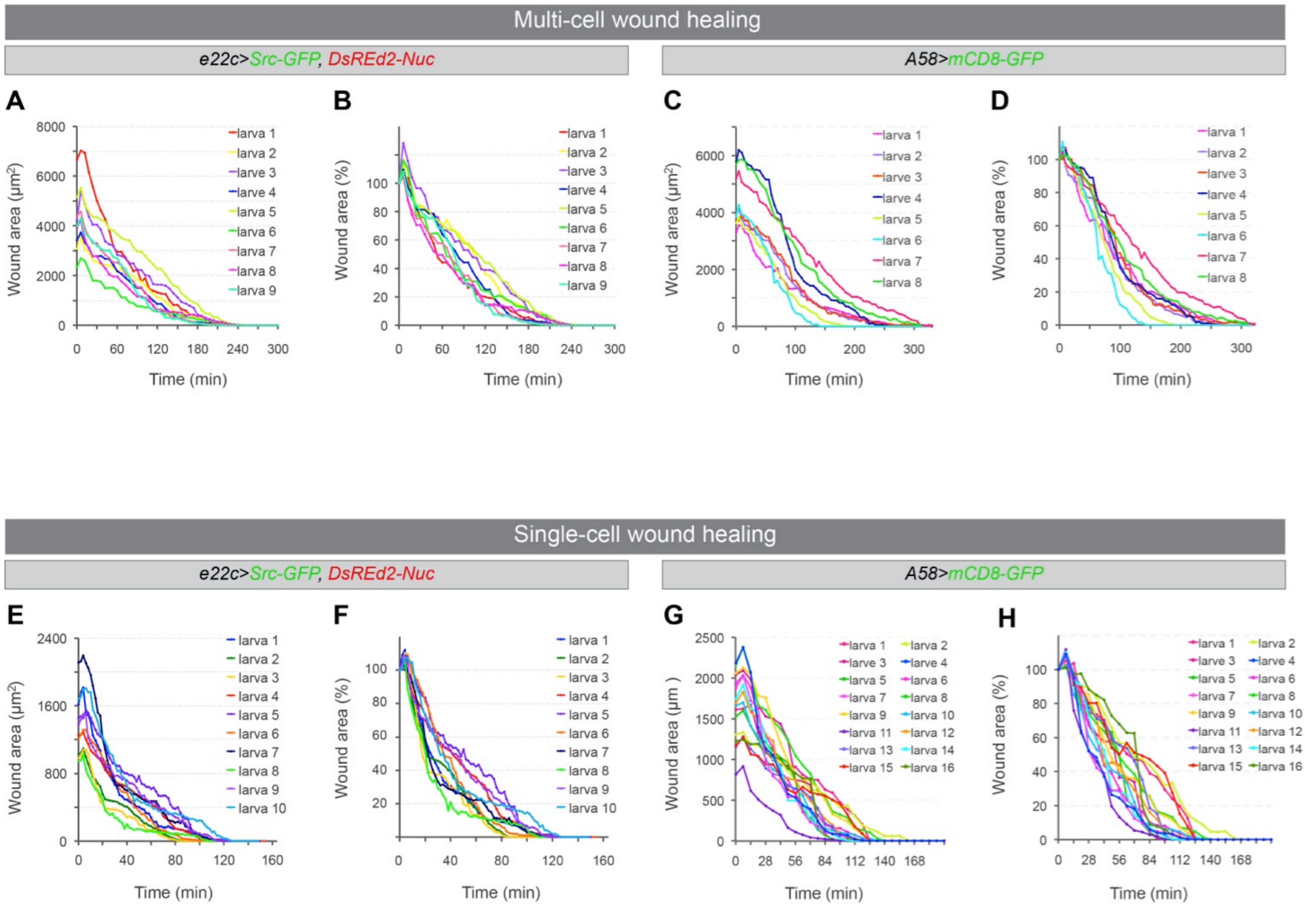

Supplementary Figure 2| Kinetics of multi- and single-cell wound healing. (**A-H**) Quantitative analysis of wound healing in control larvae. (**A-B**) Quantification of multi-cell wound healing (n=9) in the larvae shown in Supplementary Figure 1E,F and single-cell wound healing (n=10) in Figure 1c,d (*e22c>Src-GFP, DsRed2-Nuc*). (**C-D**) Quantitative analysis of multi-cell wound healing (n=8 larvae) and (**G-H**) single-cell wound healing (n=16 larvae) in control larvae (*A58>mCD8-GFP)* with mCD-8-GFP as a membrane marker. (**A,C,E,G**) Changes in absolute wound area (μm^2) and (**B,D,F,H**) in relative wound area (%). Multi-cell wound size: 3-5 cells with a wounded area of ~3,000-7,000 μm^2. Single-cell wound size: 1 cell with a wounded area of ~850-2,200 μm^2

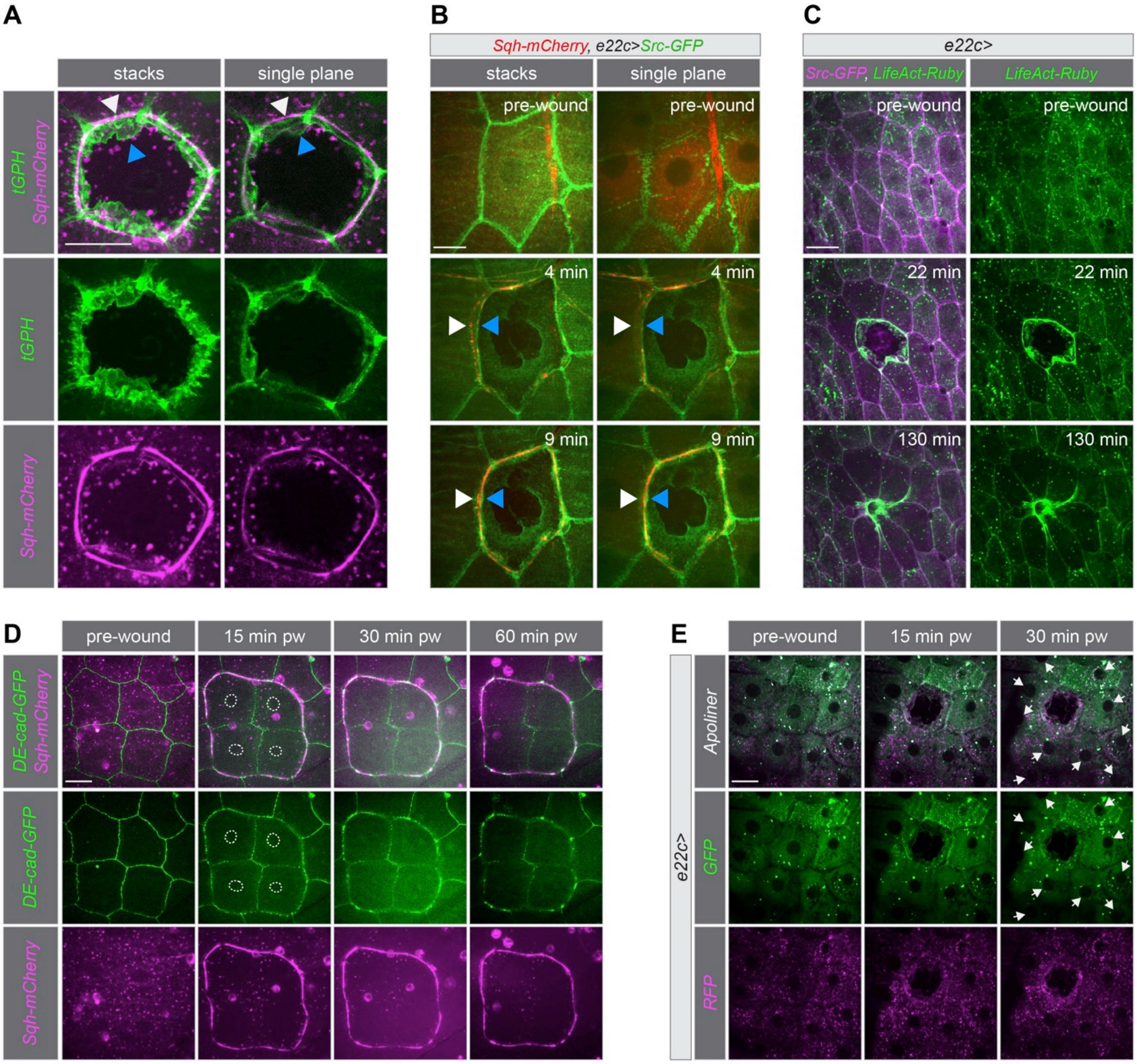

Supplementary Figure 3| Analysis of laser ablated cells for apoptosis and subcellular actomyosin cable assembly. (**A-C**) Actomyosin cable in single-cell wounds. (**A**) tGPH (green) was used to visualized plasma membrane of surrounding cells, and *Sqh-mCherry* (magenta) for the actomyosin cable. The actomyosin cable (white arrow) forms behind the lamellipodia of advancing cells (blue arrow). (**B**) Time lapse of cable formation (red). Src-GFP (green) was expressed in the epidermis (*e22c>Src-GFP*) to mark membranes (blue arrow) and *Sqh-mCherry* (red) to mark actomyosin (white arrow). (**C**) Time-lapse series of wound in larvae expressing LifeAct-Ruby (green) and Src-GFP (magenta) in the epidermis (*e22c>Src-GFP, LifeAct-Ruby*). Before wounding Sqh-mCherry is seen in a diffuse distribution as well as puncta throughout the cell. After wounding, as the cable forms, this pool is depleted in the cells directly adjacent to the wound, but not in the next row of cells. (**D**) Analysis of actomyosin cable formation in larvae expressing *DE-Cad-GFP* (green) and *Sqh-mCherry* (magenta). Four adjacent cells were killed by ablation of only their nuclei (dashed area), without damaging the lateral cell membranes. (**E**) Time-lapse series of a wound in an L3 larvae expressing the caspase sensor Apoliner[59] (*e22c>Apoliner*). No nuclear accumulation of Apoliner is seen. Scale bars: (**A-E**) 20µm.

A

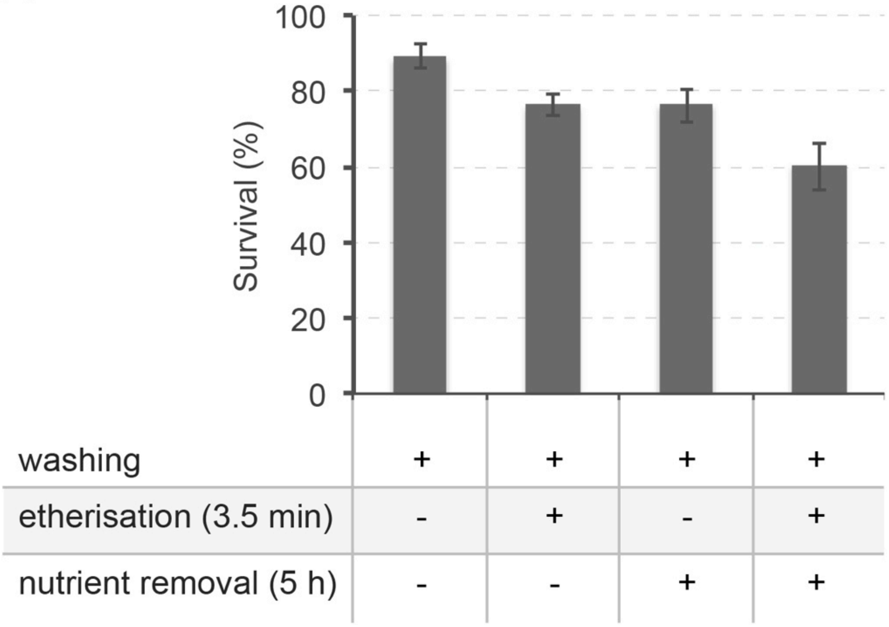

Supplementary Figure 4| Effect of immobilisation conditions on survival. (**A**) Survival rate of L3 larvae after immobilisation without laser wounding. Heterozygous *e22c>Src-GFP, DsRed2-Nuc (e22c-Gal4, UAS-Src-GFP, UAS-DsRed2-Nuc/*+) larvae were washed and immobilised with ether for 3.5 min, or washed and starved for 5h without water and nutrients in an imaging dish, or both. Average of the number of survivors as adult flies for each case: 89% after washing, 76% after washing+ether treatment, 77% after washing+starvation and 61% after combined treatment (washing+ether treatment+starvation). This experiment was done in triplicate with 100 larvae in each case. Data are given as mean ± s.e.m..

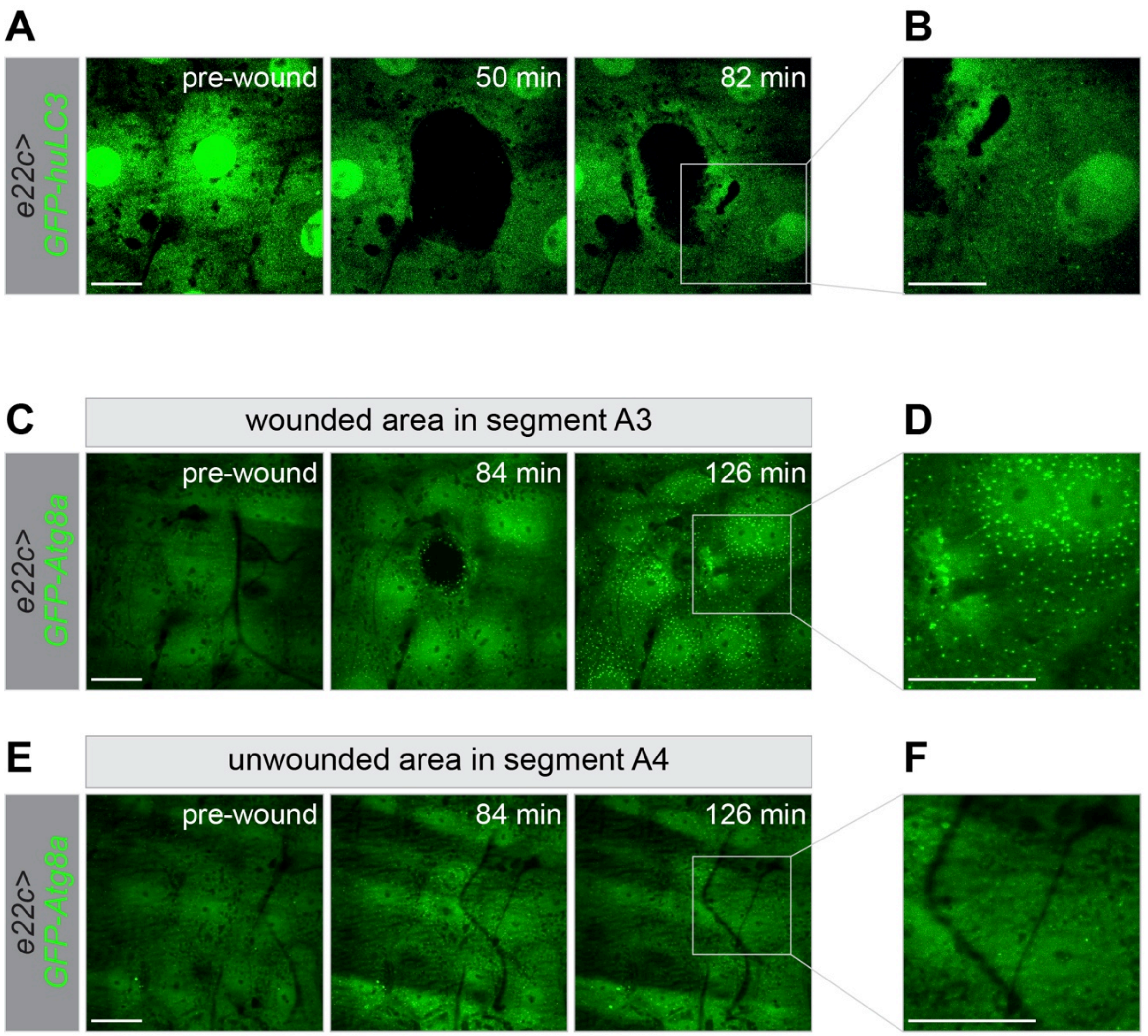

Supplementary Figure 5| Effect of imaging on induction of autophagy. (**A-B**) Time lapse images of the autophagosomal membrane marker, huLC3, during wound healing. *huLC3* was expressed ubiquitously in the epidermis (*e22c>GFP-huLC3*). GFP puncta appeared towards the end of wound closure (80±5 min). (**B**) Higher magnification of areas in grey box in **A.** (**C-F**) Time-lapse of epidermis in wounded segment A3 (**C-D**) and non-wounded segment A4 (**E-F**) of the same larva (distance ~18-20 cell diameters from the wound). Analysis of autophagy using *mCherry-GFP-Atg8a* expressed under the control of *e22-Gal4*. Only the green channel is shown. (**C**) GFP concentrated in puncta towards the end of wound closure (80±5 min) in cells around the wound and up to 4 cell diameters away. (**D**) Higher magnification of the areas in grey box in **C**. (**E**) In the non-wounded area no accumulation of green spots was observed. (**F**) Higher magnification of areas grey box in **E**. n=3 larvae. Scale bars: (**CA-B**) 10µm and (**C-F**) 30µm.

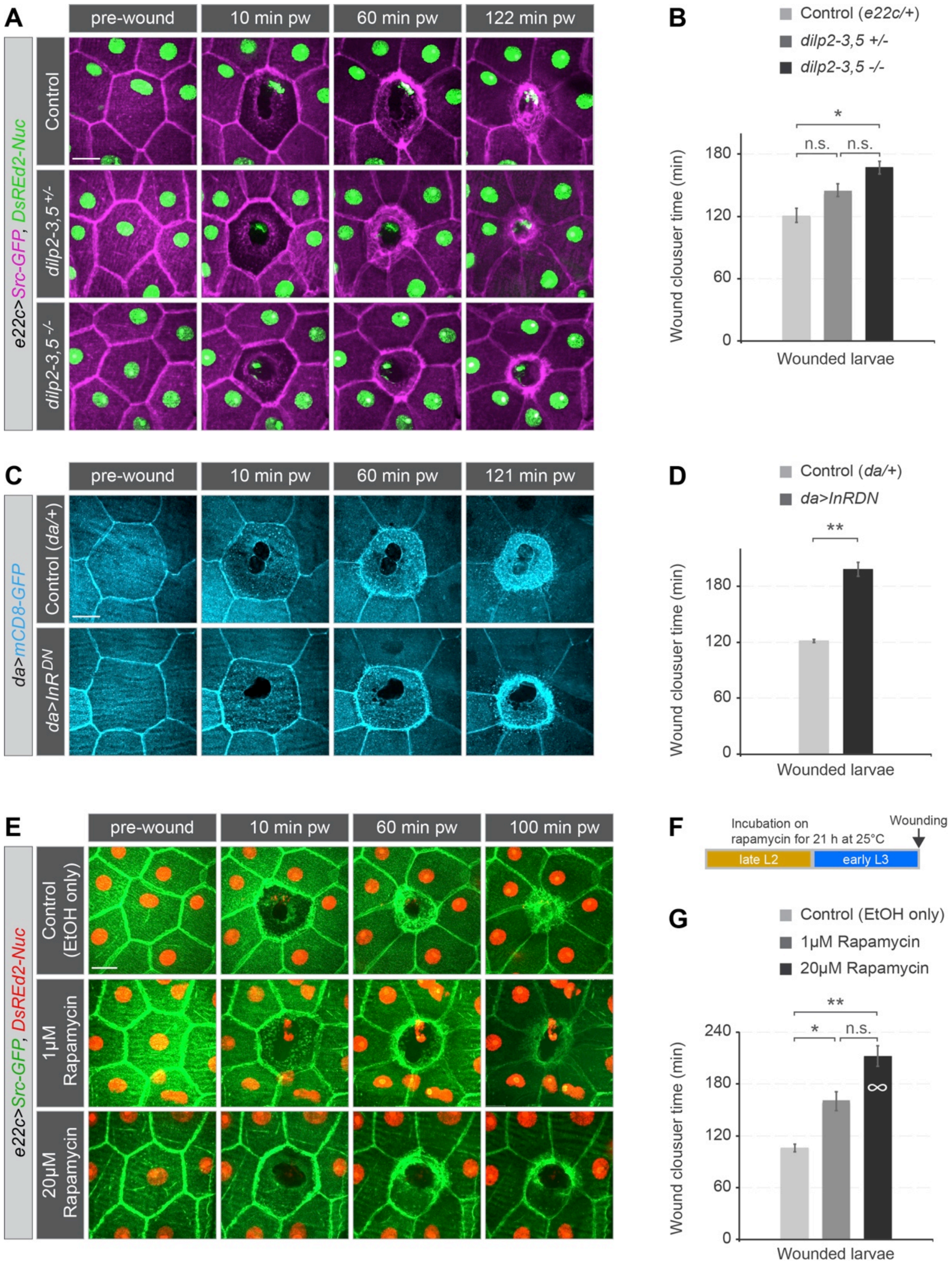

Supplementary Figure 6| Effect of systemic reduction of insulin and TOR signalling on wound healing. (**A**) Time-course of wound healing in homozygous *dilp2, dilp3*, *dilp5* triple mutant (*dilp2-3,5 -/-*), heterozygous *dilp2-3,5 +/-* and control larvae. The larvae also expressed Src-GFP and DsRed2-Nuc. (**B**) Average time of wound closure for genotypes shown in **A**: 121 min for controls, 146 min for *dilp2-3,5 +/-* and 167 min for *dilp2-3,5 -/-*. (**C**) Time-course of wound healing in larvae with ubiquitous expression of InR^{DN}. (**D**) Average time point of wound closure for genotypes shown in **C**: 122 min for control and 198 min for $da{>}InR^{DN}$ larvae. (**E**) Time-lapse series of wounds in late L2 larvae raised on food containing 0, 1 or 20 µM rapamycin in EtOH for 21 h before wounding. (**F**) Schematic for rapamycin treatment (see Methods). (**G**) Average time point of wound closure in **e**: 107 min for control, 160 min for 1 µM rapamycin and 212 min for 20 µM rapamycin. ∞: 43% of the wounds treated with 20µM rapamycin were not closed by 4 hours, and were not included in this quantification, which therefore represents an underestimate of the defects. (**B,D,G**) n=3-6 larvae each genotype or each case, mean ± s.e.m., (**B,G**) one-way ANOVA with post hoc test *p<0.01, **p<0.001, (**D**) two tailed Student's *t*-test **P<0.005. Scale bars: (**A,C,E**) 20 µm.

Transgene genotypes of larvae: (**A**) Control (*e22c-Gal4, UAS-Src-GFP,UAS-DsRed2-Nuc/+*). (**B**) Control (*da-Gal4/+; UAS-mCD8-GPF*) and $da{>}InR^{DN}$ (*da-Gal4; UAS-*InR^{DN}*, UAS-mcD8-GFP*). (**E**) *e22c-Gal4, UAS-Src-GFP,UAS-DsRed2-Nuc/+*.

A

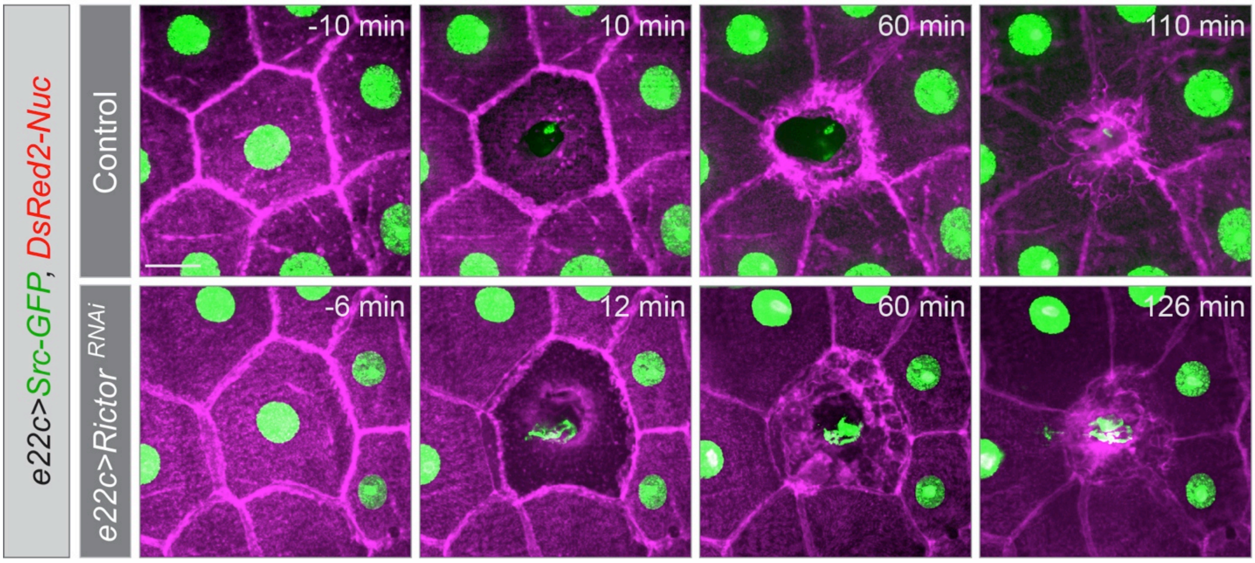

Supplementary Figure 7| Effect of TORC2 on wound healing rates. (**A**) Wound healing in L3 larvae with modified Rictor activity. All lines carried *UAS-Src-GFP* (green) and *UAS-DsRed2-Nuc* (red). (**A**) Time-course of wound healing in control *e22c/+* (*e22c-Gal4/+*) and *e22c>Rictor*^{RNAi} (*e22c-Gal4, UAS-Rictor*^{RNAi}) larvae. Reduction of Rictor did not change the course of wound healing (n=5 larvae each genotypes).

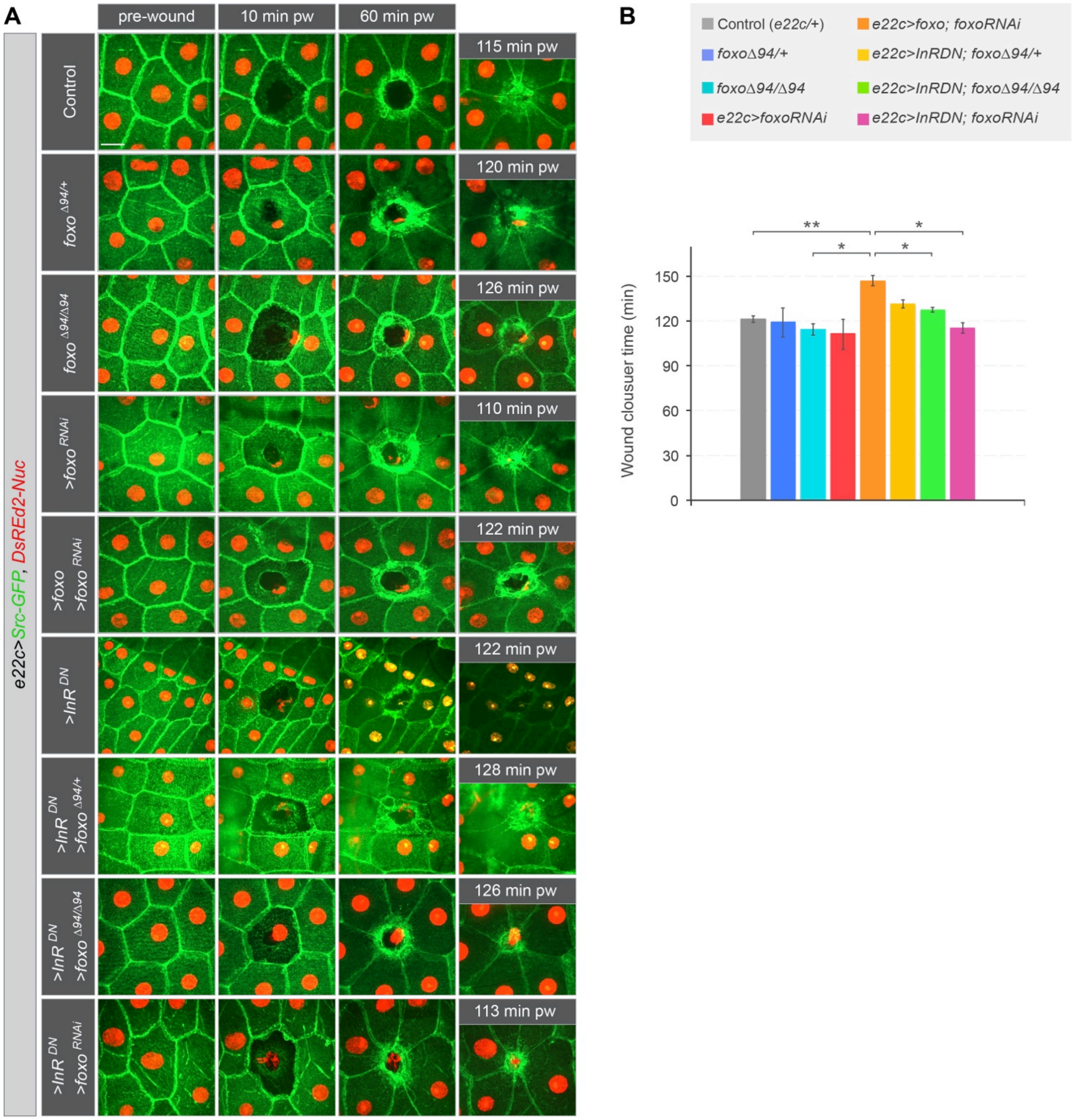

Supplementary Figure 8| Loss of FOXO in larvae expressing InR^{DN} in the epidermis. (**A-B**) Wound healing in larvae with modified InR and FOXO activity. In addition to *InR* and *foxo* constructs all larvae carried *UAS-Src-GFP* (green) and *UAS-DsRed2-Nuc* (red). (**A**) Time-lapse images of wound healing in L3 larvae. (**B**) Average time of wound closure for genotypes shown in **A**: 121 min for control, 120 min for *foxo*$^{\Delta 94/+}$, 115 min for *foxo*$^{\Delta 94/\Delta 94}$, 112 min for *>foxo*^{RNAi}, 147 min for *>foxo, foxo*^{RNAi}, *>InR*^{DN} (death during wound healing), 132 min for *>InR*^{DN}, *foxo*$^{\Delta 94/+}$, 128 min for *>InR*^{DN}, *foxo*$^{\Delta 94/\Delta 94}$ and 116 min for *>InR*^{DN}, *foxo*^{RNAi} larvae. Mean ± s.e.m. one-way ANOVA with post hoc test *p<0.006, **p<0.0006, n=3-5 larvae each genotype. Scale bar: (**A**) 20 µm.
Genotypes: Control (*e22c-Gal4/+*), *foxo*$^{\Delta 94/+}$ (*e22e-Gal4, foxo*$^{\Delta 94}$/+), *foxo*$^{\Delta 94/\Delta 94}$ (*e22c-Gal4, foxo*$^{\Delta 94}$/ *foxo*$^{\Delta 94}$), *>foxo*^{RNAi} (*e22e-Gal4, UAS-foxo*^{RNAi}), *>foxo, foxo*^{RNAi} (*e22e-Gal4, UAS-foxo, UAS-foxo*^{RNAi}), *>InR*^{DN} (*e22c-Gal4, UAS-InR*^{DN}), *>InR*^{DN}, *foxo*$^{\Delta 94/+}$ (*e22e-Gal4, UAS-InR*^{DN}, *foxo*$^{\Delta 94}$/+), *>InR*^{DN}, *foxo*$^{\Delta 94/\Delta 94}$ (*e22e-Gal4, UAS-InR*^{DN}, *foxo*$^{\Delta 94}$/*foxo*$^{\Delta 94}$) and *>InR*^{DN}, *foxo*^{RNAi} (*e22e-Gal4, UAS-InR*^{DN}, *UAS-foxo*^{RNAi}).

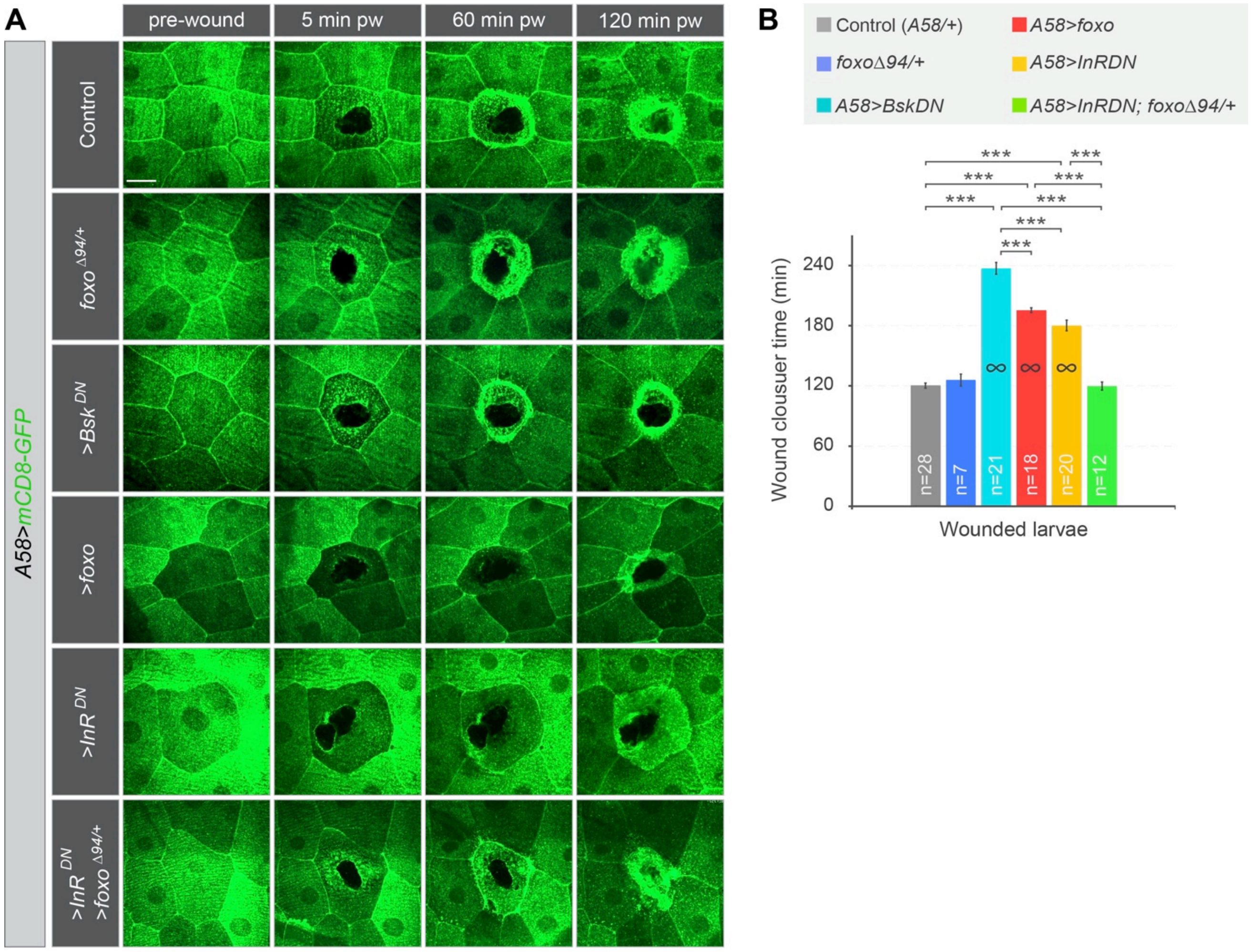

Supplementary Figure 9| Effect of loss of FOXO in cells expressing InR$^{\mathbf{DN}}$. (**A-B**) Wound healing kinetics in larvae with modified InR and FOXO activity. In addition to *InR*$^{\mathbf{DN}}$ and *foxo* constructs all larvae carried *UAS-mCD8-GFP* (green) to visualise the cell membrane. A dominant negative version of Drosophila JNK, Basket (Bsk^{DN}), a signalling molecule required for wound healing[12], was expressed in the epidermis (*A58>Bsk*^{DN}) as a control (**A**) Time-lapse images of wound healing in L3 larvae. (**B**) Average time of wound closure for genotypes shown in **A**: 120 min for control, 126 min for *foxo*$^{\Delta 94/+}$, 237 min for *>Bsk*^{DN}, 195 min for *>foxo*, 180 min for *>InR*^{DN}, 120 min for *>InR*^{DN}, *foxo*$^{\Delta 94/+}$ larvae. Mean ± s.e.m., one-way ANOVA with post hoc test ***p<0.00008, "n" is the number of larvae analysed for each genotype. ∞: The following percentages of wounds failed to close after 4 hours of imaging in these genotypes and were not included in the numerical quantification: *>Bsk*^{DN}: 52%, *>foxo*: 17% and *>InR*^{DN}: 5%. Scale bar: (**A**) 20 µm.

Genotypes: (**A-B**) Control (*A58-Gal4/+*), *foxo*$^{\Delta 94/+}$ (*A58-Gal4, foxo*$^{\Delta 94}$/+), *>Bsk*^{DN} (*A58-Gal4, UAS-Bsk*^{DN}), *>foxo* (*A58-Gal4, UAS-foxo*), *>InR*^{DN} (*A58-Gal4, UAS-InR*^{DN}) and *>InR*^{DN}, *foxo*$^{\Delta 94/+}$ (*A58-Gal4, UAS-InR*^{DN}, *foxo*$^{\Delta 94}$/+).

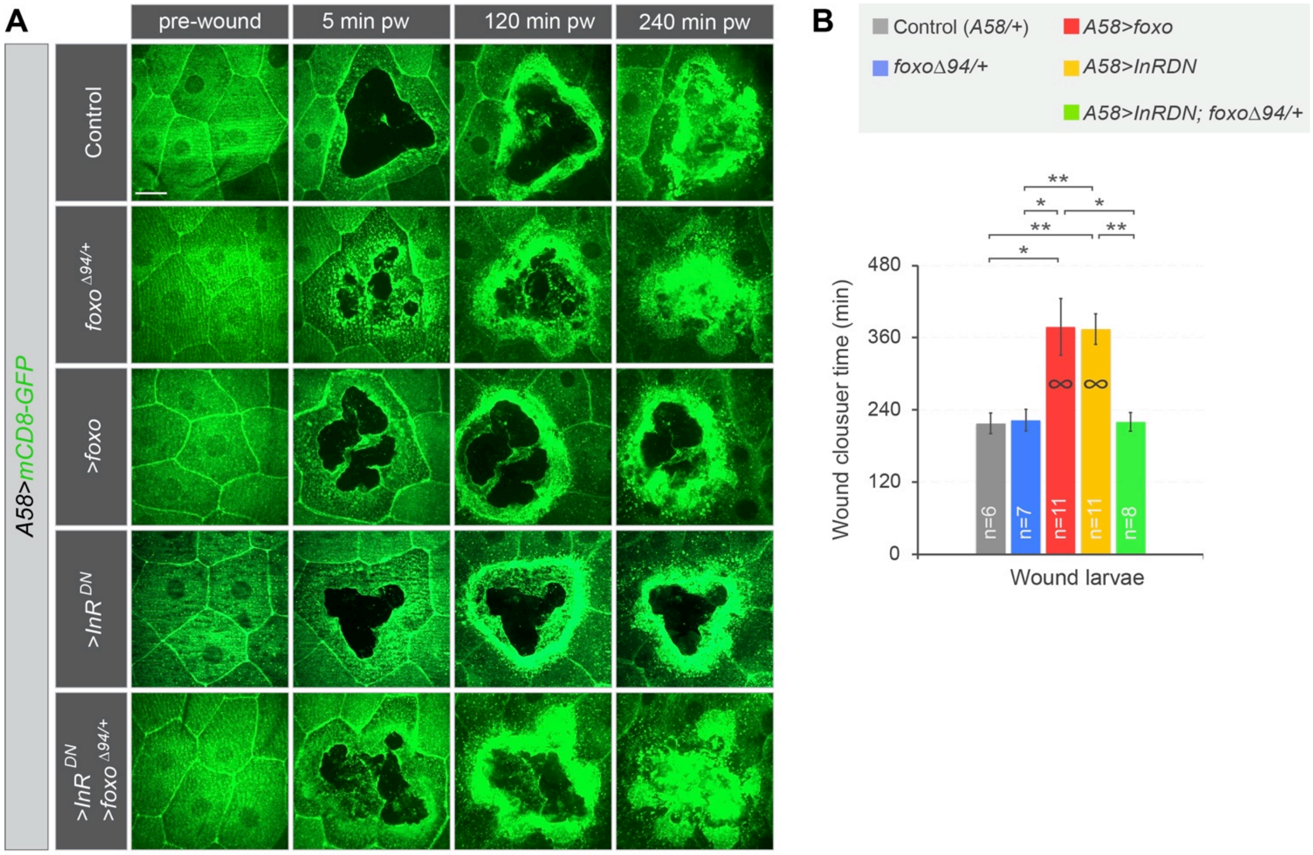

Supplementary Figure 10| Effect of loss of FOXO in cells expressing InR^{DN} in larger wounds. (**A-B**) Wound healing in larvae with modified InR and FOXO activity. In addition to *InR* and *foxo* constructs all larvae carried *UAS-Src-GFP* (green) and *UAS-DsRed2-Nuc* (red). (**A**) Time-lapse images of wound healing in L3 larvae. (**B**) Average time of wound closure for genotypes shown in **A**: 218 min for control, 223 min for *foxo*$^{\Delta 94/+}$, 378 min for *>foxo*, 374 min for *>InR*^{DN} and 221 min for *>InR*^{DN}*, foxo*$^{\Delta 94/+}$ larvae. Mean ± s.e.m., one-way ANOVA with post hoc test *p<0.01, **p<0.001 and ***p<0.0001, “n” is the number of larvae analysed for each genotype. ∞: 64% of wounds in *>foxo* larvae and 82% in *>InR*^{DN} larvae remained open after 7 hours of imaging and are not included in the quantification. Multi-cell wound size: 3-6 cells with a wounded area of ~3,000-8,500 μm^2. Scale bar: (**a**) 20 μm.

Genotypes: (**A-C**) Control (*A58-Gal4/+*), *foxo*$^{\Delta 94/+}$ (*A58-Gal4, foxo*$^{\Delta 94}$/+), *>foxo* (*A58-Gal4, UAS-foxo*), *>InR*^{DN} (*A58-Gal4, UAS-InR*^{DN}) and *>InR*^{DN}*, foxo*$^{\Delta 94/+}$ (*A58-Gal4, UAS-InR*^{DN}*, foxo*$^{\Delta 94}$/+).

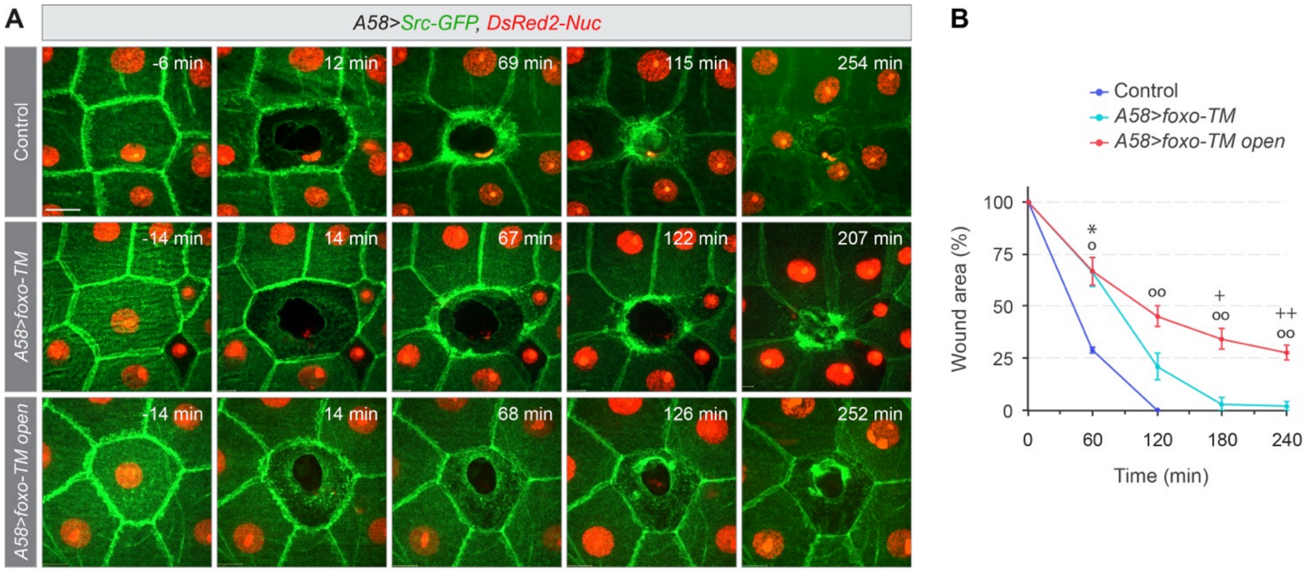

Supplementary Figure 11| Effects of raised nuclear FOXO levels on wound healing. (A-B) Wound healing in larvae with increased nuclear FOXO levels. In addition to *foxo-TM* constructs all larvae carried *UAS-Src-GFP* (green) and *UAS-DsRed2-Nuc* (red). Control (*A58-Gal4/+*) and *A58>foxo-TM* (*A58-Gal4, UAS-foxo-TM*) larvae were maintained at 18 °C before wounding and after wound healing. (**A**) Time-lapse images of wound healing in L3 larvae. (**B**) Kinetics of wound closure. Time course of changes in relative wound area. In six of nine *A58>foxo-TM* larvae the wounds remained open after 4 hours of imaging; these were quantified separately (*A58>foxo-TM* open). Mean ± s.e.m. one-way ANOVA with post hoc test, *p<0.02 (* marks for control vs. *A58>foxo-TM*), °p<0.02, °°p<0.002 (° marks for control vs. *A58>foxo-TM* open) and +p<0.02, ++p<0.02 (+ marks for *A58>foxo-TM* vs. *A58>foxo-TM* open). n=3 larvae each genotype or group. Scale bar: (**A**) 20 µm.

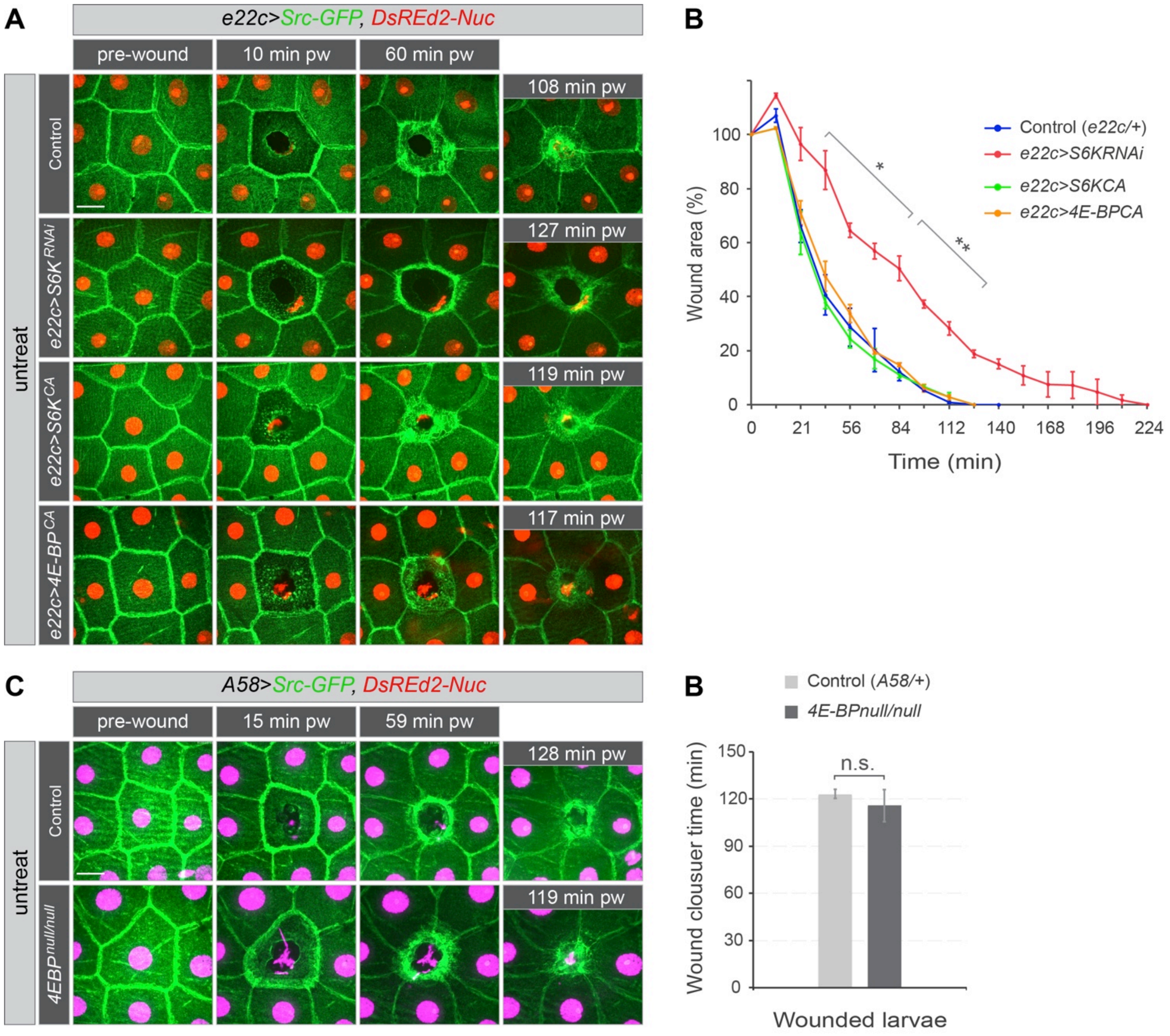

Supplementary Figure 12| TORC1 and S6K affect wound healing rates. (**A-D**) Wound healing in L3 larvae with modified S6K and 4E-BP activity. All lines carried *UAS-Src-GFP* (green) and *UAS-DsRed2-Nuc* (red). (**A-B**) Wound healing in larvae expressing RNA interference against *S6K* (*S6K^{RNAi}*), constitutively active *S6K* (*S6K^{CA}*) or constitutively active *4E-BP* (*4E-BP^{CA}*) in the epidermis under control of *e22c-Gal4*. (**C-D**) Wound healing in homozygous *4E-BP null allele* (*4E-BP$^{null/null}$*) and control larvae. (**A,C**) Time-lapse images of wound healing. (**B**) Time course of changes in relative (%) wound area for genotypes shown in **A**. Mean ± s.e.m., one-way ANOVA with post hoc test *$p<0.01$ and **$p<0.001$ n=3-5 larvae each genotype. (**D**) Average time of wound closure for genotypes shown in **C**: 123 min for controls and 118 min for *4E-BP$^{null/null}$*. Mean ± s.e.m., two tailed Student's *t*-test, n.s.: not statistically significant n=3 larvae each genotype. Scale bars: (**A,C**) 20 µm. Genotypes: (**A,B**) Control (*e22c-Gal4/+*), *e22c>S6K^{RNAi}* (*e22c-Gal4; UAS-S6K^{RNAi}*), *e22c>S6K^{CA}* (*e22c-Gal4; UAS-S6K^{STDETE}*) and *e22c>4E-BP^{CA}* (*e22c-Gal4; UAS-4E-BP^{TA}*). (**C,D**) Control (*A58-Gal4/+*) and *4E-BP$^{null/null}$* (*4E-BPnull/4E-BPnull; A58-Gal4/+*).

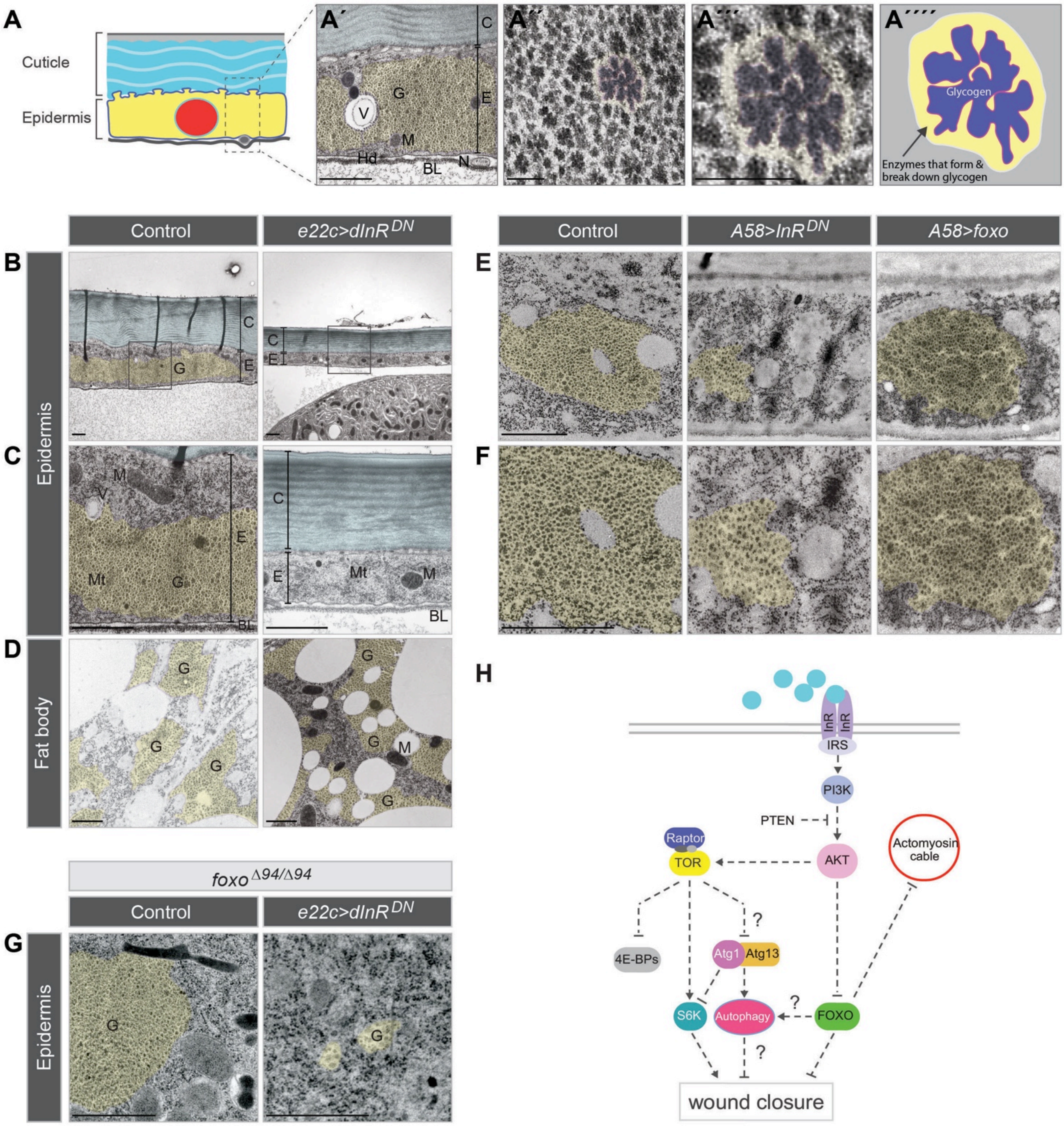

Supplementary Figure 13| Glycogen in the larval epidermis. (**A**) Cartoon illustrating the structure of the larval epidermis. (**A´-G**) Transmission electron microscopy (EM) in larvae with altered InR and FOXO activity. (**A´-G**) All larvae carried *UAS-Src-GFP* and *UAS-DsRed2-Nuc* in background. (**A´**) Normal (*e22c-Gal4/+*) epidermis with a monolayer of flattened cells on a basal lamina and a high density of glycogen. Areas with glycogen bodies in the cytoplasm are marked in yellow in all panels, and chitin-rich stratified cuticle is marked in cyan. (**A´´,A´´´**) Higher magnification showing highly branched glycogen bodies. (**A´´´´**) Cartoon illustrating a highly branched glycogen body surrounded by a layer of enzymes (yellow) that form and break down the glycogen. (**B**) Epidermis of control (*e22c-Gal4/+*) and *e22c>dInR^{DN}* (*e22c-Gal4, UAS- InR^{DN}*) larvae. (**C**) Magnification of black box in **B**. (**D**) Areas with glycogen in fat bodies of control and *e22c>dInR^{DN}* larvae. (**E-F**) Epidermis of control (*A58/+*), *A58>InR^{DN}* (*UAS-InR^{DN}*, *A58-Gal4*) and *A58>foxo* (*UAS-foxo*, *A58-Gal4*) larvae. (**F**) Higher magnification of **E**. (**G**) Epidermis of control (*e22c-Gal4/+; foxo$^{\Delta94}$/foxo$^{\Delta94}$*) and e22c>*InR^{DN}* (*e22c-Gal4, UAS-InR^{DN}; foxo$^{\Delta94}$/foxo$^{\Delta94}$*) larvae both with homozygous *foxo* deletion (*foxo$^{\Delta94/\Delta94}$*). 3-4 different sections of each larva (n=3-4 larvae each genotype) were analysed. Scale bars: (**A´**and **B-G**) 1μm and (**A´´-A´´´**) 100nm. BL: basal lamina; C: cuticle; E: epidermis; G: glycogen; Hd: hemidesmosome; M: mitochondria; Mt: microtubules; N: neuron (axon) V: vesicle (**H**) Scheme of the IIS/TOR signalling network during wound healing in the epidermis of Drosophila larvae.

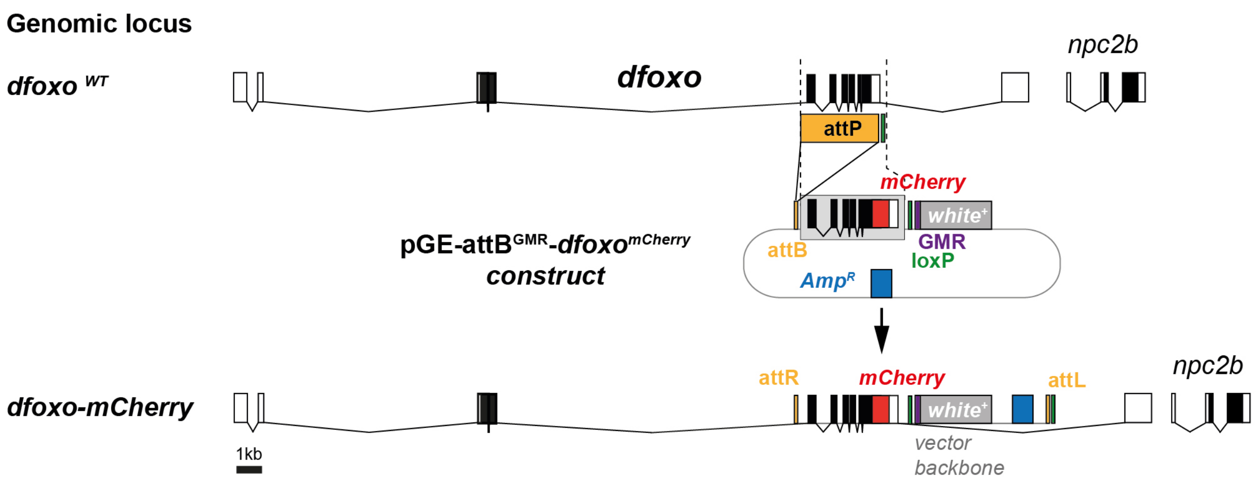

Supplementary Figure 14| Genomic engineering strategy to generate *dfoxo*-mCherry flies. Top panel: Ends-out homologous recombination[19] was used to introduce an attP site into the endogenous *dfoxo* locus, replacing indicated part of the endogenous gene. Lower panel: Subsequently, the pGE-attB^{GMR}-*dfoxo-mCherry* construct was used to insert the *dfoxo-mCherry*. GMR: Glass Multiple Reporter (an eye-specific promotor).

SUPPLEMENTARY TABLE

Table 1. Kinetics of wound closure in multi- and single-cell wounds

	**Multi-cell wounds**		**Single-cell wounds**	
	Wound area (%)	Duration to closure (min)	Wound area (%)	Duration to closure (min)
Pre wounding	100 %	0	100 %	0
Expansion phase	112±3 %	6±1 min	107±3 %	6±1 min
Early phase of closure	50±4 %	72 min	50±4 %	23 min
Late phase of closure	0	182 min	0	92 min

Wound areas were measured in *e22c-Gal4, UAS-Src-GFP, UAS-DsRed2-Nuc/*+ wounds as described in Methods.
